# Effect of iron-folic acid supplementation on change of hemoglobin among visceral Leishmaniasis patients in northwest Ethiopia: a retrospective follow up study

**DOI:** 10.1186/s12878-018-0123-2

**Published:** 2018-09-21

**Authors:** Tadele Mulaw, Amare Tariku, Adino Tesfahun Tsegaye, Zegeye Abebe

**Affiliations:** 1 University of Gondar Specialized Hospital, Gondar, Ethiopia; 2 Department of Human Nutrition, Institute of Public Health, College of Medicine and Health Sciences, University of Gondar, Gondar, Ethiopia; 3 Department of Epidemiology and Biostatistics, Institute of Public Health, College of Medicine and Health Sciences, University of Gondar, Gondar, Ethiopia

**Keywords:** Anemia, Visceral Leishmaniasis, Iron-folic acid supplementation and change of hemoglobin

## Abstract

**Background:**

An individual with visceral Leishmaniasis (VL) commonly present with anemia and one of the VL treatment center in northwest Ethiopia has been recommended iron-folic acid supplementation to these patients. But there is no documented evidence whether iron-folic acid supplementation improves the hematological profile of patients. Therefore, the study aimed to assess change in hemoglobin (Hb) and its determinant factors among VL patients with and without iron-folic acid supplementation in northwest Ethiopia.

**Methods:**

A retrospective cohort study was conducted from January 2015 to December 2016. Data were entered into Epi-Data version 3.1 and transferred to Statistical Package for Social Science (SPSS) version 20 for analysis. Independent sample T-test and linear regression were used to compare the change in Hb and identify factors associated with a change in Hb, respectively. A 95% confidence level and *p*-values less than 0.05 were used determine statistically significant.

**Results:**

From a total of 602 VL patients, 299 (49.7%) were from University of Gondar hospital. The mean (±SD) change of Hb from baseline to end of treatment was 0.99(±1.64) and 1.61(±1.88) g/dl with and without iron-folate supplementation, respectively, with mean difference 0.62, 95% CI (0.34, 0.90) and a *p*-value of < 0.0001. In multiple linear regressions, combination therapy of sodium stibogluconate-paramomycin (SSG-PM) was positively associated with a change of Hb (β [SE, p]: 0.710/0.15, < 0.0001). Whereas age (− 0.030/0.009, 0.001), nasal bleeding (− 0.261/0.123, 0.035), baseline white blood cell (− 0.139/0.044, 0.002) and hemoglobin (− 0.513/0.031, < 0.0001), end of treatment spleen size (− 0.059/0.015, < 0.0001) and iron-folic acid supplementation (− 0.574/0.163, < 0.0001) were negatively associated with change of Hb.

**Conclusion:**

Iron-folic acid supplementation had a negative effect on the change of Hb. A combination therapy of SSG-PM, age, nasal bleeding, baseline white blood cells and Hb, and iron-folic acid supplementation were the determinants of change of Hb. Therefore, avoiding iron-folic acid supplementation and strengthening VL treatment with a combination of SSG-PM and, and early identification of complications is recommended for a better outcome.

## Background

Anemia is a condition characterized by a decrease in hemoglobin concentration below the acceptable range [1]. It is a major global public health problem, especially in developing countries. According to the World Health Organization (WHO) estimate, it affects near two billion, one-quarter of the global population. Among this, Iron deficiency anemia (IDA) is responsible for approximately 50% of all types of anemia and 800, 000 deaths per year worldwide [2]. The consequences of anemia are numerous, including negatively affect cognitive performance and motor development leading to fatigue, low productivity, reduced work capacity and cognitive function, retarded physical growth, and impaired school performance [3].

In addition to nutritional deficiency, anemia is caused by infectious diseases. For instance, it is caused by one of a vector born and neglected protozoal infection, Leishmaniasis. The pathology of the disease depends on the Leishmania species, and visceral leishmaniasis (VL), caused by *Leishmania donovani andLeishmania infantum*, is the most severe and fatal with 40,000 s death annually [4, 5]. The disease is affecting largely the poorest of the poor, and highly associated poor health care systems [6].

Globally, more than 90% of VL cases occurred in six countries, including Brazil, Ethiopia, India, Bangladesh, South Sudan and Sudan [7]. Sub-Saharan Africa remains the region with the second highest incidence of VL in the world, 15% of the global burden, next to India [8]. The country Ethiopia bears the second higher incidence in East Africa, with 2000–4500 new cases per year. Individuals with VL present with anemia, hepatosplenomegaly, fever, nasal bleeding, discoloration of the skin of the hands, feet, abdomen, and face, which gave the name “kala-azar,” or “black disease” to the condition. But anemia, normocytic normochromic anemia, is a typical symptom during VL primarily caused by three mechanisms; blood loss, RBC destruction, and hemolysis, although micronutrient deficiency, particularly iron, folic acid, and vitamin B12 plays a major role in developing anemia [9–11]. In addition, duration of illness, concomitant diseases, intestinal helminths and nutritional status play a further contributory role for anemia [12].

Though countries as whole have been accelerating in reducing the incidence of VL, including developing the national guideline, early diagnosis and treatment, reservoir and vector control, and vaccination, however, functioning control programmes are not sufficient and disparities exist in the incidence of VL across countries.

Hematological improvement is expected within 2 weeks and complete recovery within 4 to 6 weeks after initiation of VL treatment weeks [13]. In northwest Ethiopia, the Abdurafi Medicine Sans Frontiers Holland Kala-azar treatment center (MSF-KTC) has been recommended iron and folic acid supplementation for those patients with VL. But the other treatment center, University of Gondar Leishmaniasis Research and Treatment Center (UoG-LRTC), didn’t supplement iron-folic acid. In addition, there is also inconclusive evidence regarding the effect of iron and folic acid supplementation on change of hemoglobin [14, 15]. Therefore, the study aimed to answer the question whether iron and folic acid have a better improvement for anemia among VL patients or not.

## Methods

### Study design and setting

An institution based retrospective cohort study was conducted from March to April 2017 at the University of Gondar Leishmaniasis Research and Treatment Center (UoG-LRTC) and Abdurafi Medicines sans Frontiers Holland kala-azar treatment center (MSF-KTC), northwest Ethiopia. This part of the country is main endemic foci for visceral leishmaniasis, including, Addis zemen, Metema, Quara, West Armachiho, Humera, and Ethio- Sudan border [16].

Both treatment centers are the larger as well as specialized leishmaniasis diagnosis and treatment centers in this region. In addition, both are supported by the non-governmental organization’s Drugs for Neglected Diseases initiative (DNDi) and Médecins sans Frontières (MSF), respectively. Each center has its own admission ward, pharmacy, and laboratory room, where routine hematological and clinical chemistry tests are done.

Admitted patients with VL in both treatment centers are routinely evaluated and the findings are documented in their respective chart. The patient’s hematology, blood chemistry, and other investigations are determined as part of other clinical evaluation modalities. Nutritional assessment, such as weight, and Body mass index are measured at admission, weekly and at discharge. In addition to VL treatment, Abdurafi MSF-KTC has been giving Iron and folic acid supplementation (IFS) to VL patients. But UoG-LRTC has not recommended iron and folic acid (NIFS) supplementation. MSF-KTC has a well-established database system for archiving patient information, whereas UoG-LRTC has a chart archiving room to keep it safe. Hence, we retrieved the data from both treatment centers easily.

### Sample size and sampling procedure

All adults (age ≥ 18 years) VL patients completed anti-leishmaniasis treatment from 2015 to 2016 were included in the study. But patients co-infected with HIV, received a blood transfusion, pregnant women, prolonged hospitalization (> 20 days) and discharged before 15 days of VL treatment were excluded. The size of the study was determined using the following assumption; 95% level of confidence, 80% power, a ratio of 1∶1 and the mean (±SD) hemoglobin of non-exposed and an exposed group were 10.5 and 9.9, ±2.6, respectively. Finally, by considering 10% incompleteness a size of 196 was obtained from each group. But we included all VL patients who fulfilled the inclusion criteria to increase the power.

### Data collection tool and procedure

A structured data extraction tool was adopted from the national VL treatment guideline and the data were extracted by four data collectors. All data collectors have a rich experience in data management and handling and they are also trained in Good Clinical Practice (GCP). In addition, one-day training was given to data collectors with practical demonstration. During the data collection period, a close supervision was done by the principal investigator. Appropriate feedback was given before the next data collection period.

### Operational definitions

#### Change of hemoglobin

Means either increase or decrease a unit of g/dl from the baseline patient’s hemoglobin [17].

#### Anemia

It was classified using the level of hemoglobin in g/dl. Hemoglobin status between12–16 g/dl and 13–17 g/dl were considered as normal for female and male patients, respectively. Mild anemia for females and male patients ranged between 11 and 11.9 g/dl and 11–12.9 g/dl of hemoglobin, respectively. For both sexes, a hemoglobin level of 8–10.9 g/dl and < 8 g/dl were considered as moderately and severely anemic, respectively [18].

#### Body mass index (BMI)

means weight divided by height in meter square (kg/m^2^) and classified as 18.5 to 24.5 normal; 17 to 18.49 mild; 16 to 17 moderate and less than 16 kg/m^2^ severe chronic energy deficiency [19].

#### Splenomegaly

It is defined as palpable spleen below the costal margin along the line of growth [20].

### Data processing and analysis

The data were entered into EpiData software version 3.1 and transformed to SPSS version 20 for data management and analysis. Percentages, means and standard deviations were used to present descriptive results. Baseline results were analyzed using the chi-square test for categorical variables. Independent sample T-test was used to compare the change in hemoglobin from baseline to end of VL treatment with and without iron-folic acid supplementation. The assumption of normality was checked both graphically and using Shapiro-wilk test. We used linear regression to identify possible predictors of change of hemoglobin. Variables with a *p*-value < 0.2 in simple linear regression was entered into multiple linear regression using backward methods. Finally, a 95% CI and a p-value of less than 0.05 were used to determine statistically significant.

## Results

### Study population and baseline clinical characteristics

A total of 602 VL patients’ charts were reviewed. Almost equal number of cases from each treatment centers, 299 (49.7%) from UOG-LRTC and 303 (50.3%) from Abdurafi MSF-KTC. The majority, 99.5%, and nearly half, 299 (49.7%), were males and migrant workers, respectively. In both treatment centers, young males (18–27 years) were the most affected group, 78.5% from supplemented and 72.9% from the non-supplemented group.

Regarding clinical symptoms at the time of diagnosis, overall, 296 (97.7%) reported fever, 269 (88.8%) loss of appetite, and 244 (80.5%) weight loss. Concerning their nutritional status, 72.9 and 88.6% were malnourished on admission among supplemented and non-supplemented groups, respectively (Table 1).

**Table 1:** Socio-demographic and baseline clinical characteristics of VL patients at UoG-LRTC and MSF-KTC northwest Ethiopia, 2017

Variables	Treatment facilities		*P*-value
	MSF-KTC (*n* = 303)	UoG-LRTC (*n* = 299)	
	*n* (%)	*n* (%)	
Sex			
Male	301 (99.3)	298 (99.7)	0.56
Female	2 (0.7)	1 (0.3)	
Age in years			
18–27	238 (78.5)	218 (72.9)	0.01
28–37	47 (15.5)	64 (21.4)	
38–47	12 (4)	16 (5.4)	
48–57	6 (2)	1 (0.3)	
Residence			
Stable residents	254 (83.8)	49 (16.4)	0.56
Migrant workers	49 (16.2)	250 (83.6)	
Duration of illness			
≤ 6 weeks	230 (75.9)	192 (64.2)	0.001
> 6 weeks	73 (24.1)	107 (35.8	
Fever > 2 Weeks			
Yes	296 (97.7)	287 (96)	0.47
No	7 (2.3)	12 (4)	
Abdominal swelling			
Yes	196 (64.7)	116 (38.8)	0.001
No	107 (35.3)	183 (61.2)	
Easy fatigability			
Yes	194 (64)	179 (59.9)	0.41
No	109 (36)	120 (40.1)	
Repeated attack of malaria			
Yes	35 (11.6)	119 (39.8)	0.001
No	268 (88.4)	180 (60.2)	
Weight loss			
Yes	244 (80.5)	274 (91.6)	0.001
No	59 (19.5)	25 (8.4)	
Loss of appetite			
Yes	269 (88.8)	270 (90.3)	0.52
No	34 (11.2)	29 (9.7)	
Nasal bleeding			
Yes	167 (55.1)	68 (22.7)	0.001
No	136 (44.9)	231 (77.3)	
Diarrhea			
Yes	41 (13.5)	36 (12)	0.73
No	262 (86.5)	263 (88)	
Cough			
Yes	157 (51.8)	143 (47.8)	0.28
No	146 (48.2)	156 (52.2)	
Pale conjunctiva			
Yes	230 (75.9)	198 (66.2)	0.01
No	73 (24.1)	101 (33.8)	
Splenomegally			
Yes	292 (96.4)	286 (95.7)	0.66
No	11 (3.6)	13 (4.3)	
Hepatomegally			
Yes	61 (20.1)	72 (24.1)	0.26
No	242 (79.9)	227 (75.9)	
Lyphadnopathy			
Yes	11 (3.6)	7 (2.3)	0.34
No	292 (96.4)	292 (97.7)	
Jaundice			
Yes	20 (6.6)	26 (8.7)	0.34
No	283 (93.4)	273 (91.3)	
Bilateral pitting edema			
Yes	38 (12.5)	67 (22.4)	0.001
No	265 (87.5)	232 (77.6)	
Baseline BMI			
Normal	82 (27.1)	33 (11.4)	0.001
Mild malnutrition	96 (31.7)	67 (22.4)	
Moderate malnutrition	73 (24.1)	76 (25.4)	
Severe malnutrition	52 (17.1)	122 (40.8)	

### Diagnosis mechanism and treatment modality of patients in both treatment sites

In case of the diagnosis criteria, more than 90% in Abdurafi MSF-KTC were diagnosed by serological test and case definition. Whereas, 269 (90%) in UoG-LRTC were confirmed by parasitological examination. The majority of patients, 215 (71%) and 269 (90%) in MSF-KTC and UoG-LRTC were treated with a combination of sodium stibogluconate and paramomycin (SSG-PM) injection for 17 days. At the end of treatment, 294 (97%) in IFS and 295 (98.7%) in NIFS groups were declared initial cure (Table 2).

**Table 2:** Diagnosis, management, and concomitant disease or complication of VL from 2015 to 2016 at UoG-LRTC and MSF-KTC northwest Ethiopia, 2017

Variables	Treatment facilities	
	MSF-KTC (*n* = 303)	UoG-LRTC (*n* = 299)
	*n* (%)	*n* (%)
Diagnosis		
Serological and/or clinical	283 (93.4)	30 (10)
Parasitological	20 (6.6)	269 (90)
Site of aspiration		
Spleen	17 (5.6)	220 (73.6)
Bone marrow	3 (1)	49 (16.4)
Anti leishmaniasis treatment		
SSG-PM	215 (71)	269 (90)
Ambisome	88 (29)	30 (10)
Treatment outcome		
Cured	294 (97)	295 (98.7)
Failure	9 (3)	4 (1.3)
Nutrition support		
Yes	205 (67.7)	3 (1)
No	98 (32.3)	296 (99)
Concomitant diseases/complications		
Malaria	15 (5)	31 (10.4)
Pneumonia	83 (27.4)	42 (14)
Ear infection	13 (4.3)	12 (4)
Intestinal parasite	58 (19.1)	64 (21.4)
Skin fungal infection	3 (1)	19 (6.4)
Pancreatitis	2 (0.7)	10 (3.3)
Sever Neutropenia (< 500 neutrophils count)	65 (21.5)	43 (14.4)
Others^a^	25 (8.3)	12 (4)

*SSG* sodium stibogluconate, *PM* Paramomycine

^a^ascities Hyperactive spleenomegally, upper respiratory infection, acute kidney injury, oral candidacies, hemorrhoid, sexually transmitted infection

### Hematological profile of patients at baseline and end of treatment

The overall prevalence of anemia was 303 (100%) and 300 (99%) at the baseline and end of treatment among the supplemented group, respectively. Regarding the severity of anemia, 147 (48.5%) and 90 (29.7%) of the patients had severe anemia at baseline and end of treatment. Similarly, the overall prevalence of anemia among the non-supplemented group was 291 (97.4%) and 278 (93%) at beginning and end of treatment, respectively. Concerning the hematological parameters, the mean (±SD) of white blood cells count was 3.4(±1.5) × 10^3^/μl in the supplemented and 3.8(±1.4) × 10^3^/μl non-supplemented groups at the end of treatment (Table 3).

**Table 3:** BMI and Hematological profiles of VL patients at baseline and end of treatment with and without iron and folic acid supplementation at UoG-LRTC and MSF-KTC northwest Ethiopia, 2017

Characteristics	Treatment facilities					
	MSF-KTC (*n* = 303)			UoG-LRTC (*n* = 299)		
	Initial	Final	*P*-value	Initial	Final	*P*-value
	Mean (±SD)	Mean (±SD)		Mean (±SD)	Mean (±SD)	
WBC (×10^3^/μl)	1.98 (1.4)	3.4 (1.5)	< 0.001	2.3 (1.3)	3.8 (1.4)	< 0.001
Hemoglobin	8.4 (4.7)	9.2 (1.7)	0.002	9.0 (4.8)	10.39 (1.8)	< 0.001
Mean cell volume (MCV)	79.2 (7.3)	81.0 (9.0)	< 0.001	82.0 (9.3)	85.5 (8.6)	< 0.001
BMI	17.4 (1.6)	17.9 (1.8)	< 0.001	16.5 (1.6)	16.9 (1.6)	< 0.001

*WBC* white blood cell, *IQR* Interquartile range, *SD* standard deviation, *MCV* mean cell Volume, *BMI* Body Mass Index

### Comparison of change of hemoglobin between two groups

The result of a change in hemoglobin (Hb) of VL patients with and without iron-folic acid supplementation is summarized in (Table 4). Among the supplemented group, the mean (±SD) Hb at baseline and end of treatment was 8(±1.7), and 8.99(±1.67) g/dl, respectively. Similarly, the mean (±SD) Hb at baseline and end of treatment was 8.77(±2.18) and 10.38(±1.85) g/dl among the non-supplemented group, respectively. The finding indicates that there is a statistically significant difference between supplemented and non-supplemented group within the change of Hb, *P* <  0.05.

**Table 4:** Comparison of change of Hb with and without iron-folic acid supplementation among VL patients at UoG-LRTC and MSF-KTC northwest Ethiopia, 2017

Variable	Hemoglobin (g/dl)					95% CI of the mean difference		*P*-value
Iron-folic acid supplementation	N	Initial	Final	Change of mean (±SD)	Mean difference	Lower	upper	
		Mean (±SD)	Mean (±SD)					
Yes	303	8.0 (1.7)	8.99 (1.67)	0.99 (1.64)				< 0.0001
No	299	8.77 (2.18)	10.38 (1.85)	1.61 (1.88)	0.62	0.34	0.90	

### Factors associated with a change of hemoglobin

According to multivariable linear regression analysis, age, nasal bleeding, iron-folic acid supplementation, end line spleen size, baseline WBC and Hb count were negatively associated with the change of Hb, whereas a combination of therapy of SSG-PM was positively associated with the change of Hb (Table 5).

**Table 5:** Factors associated with a change of Hb among VL patients at UoG-LRTC and MSF-KTC northwest Ethiopia, 2017

Independent variables	(B) Coefficients/std-error	95% (CI for B)		*P*-value
		Lower	Upper	
Constant	6.866/0.397	6.085	7.646	< 0.0001
SSG-PM treatment	0.710/0.15	0.416	1.005	< 0.0001
Age	−0.030/0.009	−0.049	−0.012	0.001
Nasal bleeding	−0.261/0.123	− 0.502	− 0.019	0.035
Baseline WBC	− 0.139/0.044	− 0.226	− 0.052	0.002
Baseline Hb	− 0.513/0.031	− 0.574	− 0.452	< 0.0001
End of treatment spleen size	− 0.059/0.015	− 0.088	− 0.029	< 0.0001
Iron-folic acid supplementation	− 0.574/0.163	−0.895	− 0.253	< 0.0001

## Discussion

This study was tried to evaluate the effect of iron-folic acid supplementation on change of Hb among VL patients. Accordingly, iron-folic acid supplementation had a negative effect on the change of Hb. Iron supplementation may create a conducive environment for parasite survival and growth [15]. In addition, iron has a role in modulating iron superoxide dismutase activity and hydrogen peroxide generation, which are important for parasite differentiation and increasing parasite load. Furthermore, iron availability controls the expression of Leishmania iron transporter1, resulting in iron uptake and an overall increase in intracellular iron availability and parasite load [21].

In this study, the average change of Hb was increased by 71% among patients treated with Sodium stibogluconate-paromomycin (SSG-PM) combination compared to patients treated with Ambisome alone. This finding is supported by a study finding from Sudan [22]. This explained by the treatment directly killed the parasite inside phagolysosome by inhibiting trypanothione reductase enzyme (an enzyme that recycles oxidized trypanothione to keep the trypanothione in reducing state). So, this decreased the parasite load and may contribute hematological improvement [23].

Age of the patients was one of the predictors of change of Hb. Accordingly, a year increase in age; the average change in Hb was decreased by 3%. This finding is consistent with the study conducted in East Boston [24]. This could be explained by as the age of patients advanced associated with a gradual decline in the immune system, which contribute to the severity of the disease and needs longer time to recover.

The size of spleen at the end of VL treatment was inversely related to the change in Hb among VL patients. This might be due to sequestration and destruction of red blood cells in enlarged spleen largely contribute to poor recovery from anemia [25]. In addition, blood loss, dysfunction in erythropoiesis, and up-regulated destruction of erythrocytes are the major contributory factors for anemia.

The average change of Hb was decreased by 26% among patients with nasal bleeding (β = − 0.261; 95% Cl: − 0.502, − 0.019). This could be due to very low platelet count as the result of bone marrow suppression. So that the improvement of hemoglobin might not be the same as to those did not have nasal bleeding.

In this study, Iron and folic acid supplementation decreased the average change of hemoglobin by 57% at 95% CI (− 0.895, − 0.253). This finding is consistent with a finding from Belgium which demonstrates adequate supply of iron is needed for the life cycle of Leishmania parasite. In addition, experimental studies on an animal model using iron chelating approach indicates that iron deprivation reduce the rate of promastigotes multiplication in a dose-dependent fashion and iron is also crucial for anti-oxidizing function and other metabolic reaction of the parasite which makes the replication favorable [15].

Similarly, a study conducted in Portugal, the mammalian stage of Leishmania (amastigotes), showed that the amastigotes had high affinity to iron and are also capable of exploiting iron from hemin and hemoglobin for nutritional purposes [14]. In addition, the Leishmania donovani (LD) acquire iron from the host macrophages for survival and growth through different mechanisms. Nevertheless the host macrophages resist by actively sequestering iron from the parasite, the LD directly scavenges iron and utilizes for its intracellular growth [26].

On the other hand, a study conducted in Portugal, the effects of iron supplementation or deprivation on the growth of L.infantum, confirmed iron deficiency did not affect the protozoan growth, whereas iron overload decreased its replication in the liver and spleen of a susceptible mouse strain [27].

## Conclusion

Iron-folic acid supplementation had a negative effect on change in Hb among VL patients. A combination therapy of SSG-PM, age, nasal bleeding, baseline white blood cells and Hb, and iron-folic acid supplementation were the determinants of change of Hb. Therefore, avoiding iron-folic acid supplementation and strengthening VL treatment using a combination of SSG-PM during the active infection, and early identification of complications is recommended for a better outcome.

## Abbreviations

AOR: Adjusted odds ratio
CI: Confidence interval
COR: Crude odds ratio
Hb: Hemoglobin
MSF-KTC: Abdurafi Medicine Sans Frontiers Holland Kala-azar treatment center
SD: Standard deviation
SPSS: Statistical Package for Social Science
SSG_PM: Stibogluconate-paramomycin
UoG_LRTC: University of Gondar Leishmaniasis Research and Treatment Center
VL: Leishmaniasis
WHO: World Health Organization
